# Neurobehavioral, and Cytoarchitecture Evaluation of the Therapeutic Potential of *n*‐butanol fraction of *Phoenix dactylifera L*. on lead acetate triggered neurodegenerative changes in Wistar rats

**DOI:** 10.1002/alz.088315

**Published:** 2025-01-03

**Authors:** Stephen S Lazarus, Sunday Abraham Musa, Abel Noserenme Agbon, Oladele Sunday B

**Affiliations:** ^1^ Ahmadu Bello University, Zaria, Kaduna Nigeria; ^2^ Ahmadu Bello University Zaria, Zaria, Kaduna Nigeria; ^3^ Ahmadu Bello University Zaria, Zaria, kaduna Nigeria

## Abstract

**Background:**

Lead, a pervasive and toxic environmental pollutant, of particular concern is its impact as a trigger for neurodegenerative diseases. *Phoenix dactylifera* (date palm), has garnered attention due to its pharmacological properties: antioxidant and anti‐inflammatory, attributed to its rich flavonoid content. This assessed the therapeutic potentials of *n‐*butanol fraction of *P. dactylifera* (BFPD) on the behavioral and histomorphology of hippocampus lead acetate (PbA)‐induced neurotoxicity in Wistar rats.

**Method:**

Forty‐two rats were categorized into seven groups (*n* = 7). Two experimental phases were employed: Toxicity‐phase, and Treatment‐phase. Toxicity‐phase: all rats received PbA (120 mg/kg), for 14 days, except group one (control); Treatment‐phase: group II (sacrificed), group III (Natural recovery), while groups (IV‐VII) received (500 mg/kg, 750 mg/kg and 1000 mg/kg) *n‐*BFPD and (100 mg/kg) vitamin C (as reference antioxidant). Treatment was via oral route, which lasted for 28 days. Therapeutic properties of *n‐*BFPD were assessed using Neurobehavioral assessment: Morris water maze performance and Novel object recognition test for (spatial memory, learning, and cognition); Oxidative stress biomarkers were assay using Malondialdehyde [MDA], and Superoxide dismutase [SOD], and microscopic hippocampus examination (CA1 and CA3) using histological and histochemical staining techniques and quantification of Nissl substance stain intensity using a computer running image analysis software (imageJ).

**Result:**

PbA‐treated group revealed neurodegenerative changes as remarkable (*p<0.05)* memory, learning and cognition impairment, elevation MDA levels and decrease in antioxidant enzymes (SOD), and cytoarchitectural distortions evidenced by necrotic nuclei, vacuolation and pyknosis, compared to the control. However, treatment with *n‐*BFPD revealed cognitive improvement in memory and learning; decreased MDA levels and increased SOD activities. Mild distortion‐to‐relatively normal neuronal cytoarchitecture relative to the control was also observed with *n‐*BFPD treatment.

**Conclusion:**

*n‐*BFPD possesses potential therapeutic properties against PbA‐induced neurobehavioural and cognitive deficit and pathological changes which are indicator of neurodegenerative diseases.

**Keyword**: cytoarchitecture, cognition, neurodegeneration

